# Clinical Complaints amongst Patients in a Guyanese Prison

**DOI:** 10.5539/gjhs.v4n6p47

**Published:** 2012-08-22

**Authors:** Raywat Deonandan, Jessica Wynn Lockhart, Brenna Mahony, Glenda Mindlin, Joanne Laine-Gossin, Nazmoon Audam, Louis Nel, Melissa Sissons, Bekkie Vineberg

**Affiliations:** 1 Interdisciplinary School of Health Sciences, University of Ottawa, Ottawa, Canada; 2 Ve’ahavta, Toronto, Canada; 3 Bethany School of Medical Evangelism, Bethany, Guyana

**Keywords:** epidemiology, prison, global health, volunteerism, underserved communities

## Abstract

**Background:**

Incarcerated populations are at particular risk for developing specific health conditions. Prior studies of prisons in developing countries have focused on the threat of communicable diseases, though anecdotal evidence suggests that chronic conditions are of particular concern. This study constitutes the first published investigation of health complaints offered by residents of a prison in the South American nation of Guyana.

**Method:**

In 2010, a medical team sent by the Toronto non-governmental organization Ve’ahavta visited the Mazaruni prison in the interior of Guyana. Data on patient encounters was collected as part of the triage activity.

**Results:**

Care was given to 108 patients, staff and family members. Contrary to literature expectations, 50% of complaints concerned musculoskeletal issues, while only 11% were genitor-reproductive. Upon examination, 30.6% of patients were experiencing musculoskeletal problems, most commonly back pain.

**Conclusion:**

Future medical interventions to this and comparable low- and middle-income country prisons should more vigorously consider physiotherapeutic interventions, in addition to the expected addressing of infectious diseases.

## 1. Introducation

Incarcerated populations are at particular risk for developing specific health conditions, due to living in close quarters with large numbers of people, a reduced variety of physical activities, lack of individual control by prisoners over their diets, a heightened risk of physical violence, and unique and unrelenting psychological stresses. In other words, they have disproportionate health care needs (May et al., 2010). Globally, prison-centred communicable diseases remain a significant and unaddressed health crisis (Davies, 2007). This is particularly true in low income countries (LMICs), where communicable diseases are typically more prevalent, and where global scourges, such as HIV/AIDS and TB, find ready footholds in incarceration facilities with variable hygiene and disease control standards, and with heightened rates of intravenous drug use (Dolan et al., 2007).

Most publication activity regarding health concerns in LMIC prisons focuses on the spread of those two aforementioned high profile infectious diseases. However, both published and anecdotal evidence suggest that non-communicable factors may be quite prevalent and worthy of greater attention. Specifically, mental health and musculoskeletal issues (Dumont et al., 2012) may account for a fair proportion of heretofore unmeasured suffering in incarcerated populations in LMICs.

This study describes the findings of a volunteer medical mission to the interior of Guyana, specifically the Mazaruni region, where direct clinical care was given to residents of a prison with two partnered facilities: the main Mazaruni prison, which houses repeat offenders and perpetrators of serious and violent crimes, and Sibley Hall, which is an associated facility for first offenders.

Guyana is an English-speaking country on the South American continent, but with economic and cultureal affiliations with the nations of the Caribbean. It is underpopulated, with fewer than 800,000 residents, though almost all are clustered along the Caribbean coast. The interior of the country is largely undeveloped rainforest, sparsely populated by AmerIndian tribes, and of interest to international mining and timber concerns. Guyana is considered a poor country, with high rates of HIV/AIDS, Tuberculosis and malaria, relative to most other Western hemisphere countries.

Nationwide, Guyana has approximately 2122 prisoners (a rate of 281 per 100,000 residents), of whom 96% are male (International Centre for Prison Studies, 2012). This rate is on par with that in neighbouring Brazil and French Guyana, but about 50% greater than the rates seen in Suriname and Venezuela. The incarcerated individuals are spread over a total of 5 major institutions in Guyana. Given its stated prison capacity of 1580 persons, the nation is 134% over-occupied (International Centre for Prison Studies, 2012). Guyana’s base population has been shrinking over the past few years, but its number of prisoners has been increasing sharply since at least 1995 (International Centre for Prison Studies, 2012), meaning that its rate of incarceration has been accelerating rapidly indeed.

The Guyanese prison population has not been well studied from a health perspective, even less so those incarcerated in this, more remote institution nestled on the fringes of the rainforest. While our focus was the provision of direct care, our secondary intent was to use the resulting clinical encounter data to describe the patient profile of this population, focusing on both the patients’ stated complaints and on the resulting diagnoses post-examination.

## 2. Method

In 2010, as part of a volunteer medical mission organized by the Toronto non-governmenta; organization (NGO) called Ve’ahavta, a team consisting of two physicians, two nurses, one physiotherapist, one epidemiologist, one team leader and several logistical support people undertook a one day visit to the Mazaruni prison to give direct clinical care to assembled patients originating from both the main prison and the associated lesser facility called Sibley Hall. The clinic lasted one day, and included the triage, diagnosis and care of all prisoners seeking service, as well as service provision for members of the prison staff and their families. The only lab tests that the team was equipped to perform *in situ* were tests of blood and urine sugar, and pregnancy tests.

Handwritten data was collected pertaining to patient demographics, clinical complaints and immediate diagnoses. Patient complaints were categorized into 11 broad diagnostic categories. Basic frequency analysis was applied to the resulting data, using SPSS version 17.0. Given our low sample size, further statistical testing was not deemed appropriate.

Research ethics was granted for this study by the University of Ottawa Office of Research Ethics and Integrity.

## 3. Results

A total of 108 patients were seen, of whom 6 were prison staff or their family members. Only one woman, a prison guard, was examined; this was not surprising, since only a handful of women worked in the facility. Age and weight were normally and symmetrically distributed across all subjects; our sample had a mean age of 33.7 years, and a mean mass of 71.9 kg. Blood pressure was more skewed, given the presence of several febrile subjects, but median BP was a remarkable textbook normal of 120/80.

No pregnancy tests were given to this cohort, due to the dearth of female patients. Unfortunately, the results of the blood and urine sugar tests did not survive transport back to Canada, and therefore cannot be reported herein.

General categories of patient complaints, as assessed during triage, are summarized in Table 1, while post-examination diagnoses are summarized in Table 2.

**Table 1:** Categories of patient complaints, assessed at triage

Patient complaint category	Number of patients	Percent distribution
Musculoskeletal	54	50.0
Genito-reproductive (including all STIs)	12	11.0
Head and Neurological	11	10.2
Skin (including all rashes)	7	6.5
Vision	7	6.5
Respiratory	6	5.6
Circulatory	6	5.6
Urological	5	4.6
Endocrine/metabolic (including diabetes)	4	3.7
External injury	4	3.7
Gastrointestinal	3	2.8

**Table 2:** Categories of diagnoses by physician upon examination

Diagnosis category	Number of patients	Percent distribution
Musculoskeletal	33	30.6
Tuberculosis	3	2.8
Hydrocele	3	2.8
Fungal infection	3	2.8
Hypertension	3	2.8
Diabetes	2	1.9
Folliculitis	2	1.9
Hemorrhoids	2	1.9
Vision	2	1.9
Various contusions	2	1.9
All others	1 each	<1

## 4. Discussion

Contrary to the literature’s focus on HIV/AIDS and TB, our experience was that infectious concerns were secondary to non-communicable concerns in this LMIC prison population. Musculoskeletal issues were predominant. The second most common category of complaint was genitor-reproductive in nature, though our clinical examinations typically did not result in a confirmatory diagnosis. Many genito-reproductive complaints were in fact hernia or other lower abdominal strains. Lower back pain was the most common form of musculoskeletal issue, and was frequently confounded by the effects of poor digestion of the standard prison diet of beans and rice. Only one of the 6 non-prisoners examined was suffering from a musculoskeletal issue.

Unsurprisingly, our physiotherapist was the most put upon during the clinic, having to give care in groups rather than individually, in order to manage the large number of back pain complaints. Analgesics and anti-inflammatory drugs, most commonly Ibuprofen, were prescribed at a high rate.

Incarcerated populations in high inome countries (HICs) are comparatively well studied, but those experiences do not seem applicable to LMICs. In American prisons, for example, it can be argued that the incarcerated tend to be low-income, therefore less medically well served than the American general population (Dumont et al., 2012) prior to their imprisonment. Studies on the American population also focus on HIV/AIDS, TB, Hepatitis C and other STIs, while chronic conditions like diabetes and overweight are only now emerging as issues worthy of study (Dumont et al., 2012; Wolff et al., 2012). Mental health, particularly related to addiction, has always been a matter of interest in HIC prisons (Gisin et al., 2012).

The Mazaruni prison population likely has a higher proportion of Aboriginal prisoners than do prisons elsewhere in the country, due to the comparatively higher density of Amerindian settlements in the region. Incarcerated Aboriginal populations in other countries, for example Australia, are known to exhibit greater rates of mental health symptoms than are seen in the non-incarcerated communities (Heffernan et al., 2012).

In comparison, the Guyanese experience is minimally concerned with STIs (though the country suffers from an impactful national HIV rate of 2.4% (Government of Guyana, 2012). And the omission of a mental health focus is ubiquitous across most LMICs, in both prison populations and the general public (Siriwardhana et al., 2011). But perhaps the most important distinction between the American HIC prison experience and the Guyanese LMIC experience is prisoners’ accessibility to health care. In the words of Dumont et al. (2012), “Among the ironies of contemporary social and political attitudes regarding prisoners in the United States is that the incarcerated constitute almost the only group that has a constitutional right to health care.” Whereas, Guyanese prisoners must rely upon cyclical visits from a regional doctor, as well as more infrequent, though often better equipped, visits from foreign medical teams.

This may partially explain the comparatively high rate of musculoskeletal complaints. In well resourced environments, prisoners would have received care well before back pains, for example, would not have progressed to more serious and intractable stages, and would have access to a wider variety of analgesics beyond Ibuprofen.

The implications of our findings relate to the preparedness of future medical missions to this and similar LMIC prisons. Basing our staffing and pharmacological expectations on national-level indicators would suggest that infectious diseases, primarily HIV/AIDS, STIs and TB, would be the primary health concerns of this population. What is more needed, it would seem, are greater physiotherapeutic interventions and analgesics. Liaison with prison administrators is also indicated, to perhaps investigate a wide variety of nutritional options for inmates, though this might not be feasible due to local resource constraints.
